# Review of the unprecedented impact of the COVID-19 pandemic on the occurrence of eating disorders

**DOI:** 10.1097/YCO.0000000000000815

**Published:** 2022-08-05

**Authors:** Karien Meier, Daphne van Hoeken, Hans W. Hoek

**Affiliations:** a Parnassia Psychiatric Institute, The Hague, The Netherlands; b University of Groningen, University Medical Center Groningen, Department of Psychiatry, Groningen, The Netherlands; c Columbia University, Mailman School of Public Health, Department of Epidemiology, New York, New York, USA

**Keywords:** coronavirus disease-2019, eating disorder, epidemiology, online treatment, pandemic

## Abstract

To review the recent literature on the impact of the coronavirus disease-2019 (COVID-19) pandemic on incidence and severity of symptoms of eating disorders (EDs).

**Recent findings:**

A worrying increase of EDs in- and outpatients has been reported since the COVID-19 pandemic began in 2019/2020. Restrictions implemented during the pandemic to protect populations against COVID-19 increased the risk for onset and for worsening of EDs by disrupting eating and exercise routines, social isolation, lack of support, and limited access to healthcare. Substantial increases since the start of the pandemic have been reported for overall incidence (15%), hospital admissions (48%) and emergency department visits (11%) for EDs, with even higher increases among women and children or adolescents with an ED. During the pandemic, ED patients reported increased severity of ED-specific symptoms and increased anxiety, depression and suicidal ideations and -attempts. Treatments shifted largely toward online methods for continuity of care, despite concerns about the quality of care provided and difficulties in self-monitoring. Our review provides preliminary evidence for a similar effectiveness of online treatment to prepandemic face-to-face treatment. In-person assessment remains essential for detecting EDs and for those requiring medical admission.

**Summary:**

Although the ongoing COVID-2019 pandemic affected mental health globally, research shows that it particularly affected individuals with an ED diagnosis or at risk for an ED, especially women, children and adolescents, and those with anorexia nervosa.

## INTRODUCTION

The emergence of coronavirus disease-2019 (COVID-19) has impacted almost every aspect of life for most people worldwide. The global COVID-19 pandemic has impaired mental health, especially in people with preexisting psychiatric conditions. For instance, depressive and anxiety disorders have been exacerbated during the pandemic, with 28% and 26% increases in global prevalences, respectively [1]. The incidence of other syndromes, such as posttraumatic stress disorder and burnout, has also increased, especially among medical personnel [2]. Emergency department visits for a variety of mental health conditions increased considerably during the pandemic compared to the prepandemic period, particularly among young individuals [3^▪▪^].

The pandemic has had far-reaching consequences for individuals with an ED – affecting their daily routines, social contact, and access to healthcare. Eating disorders (EDs) are disabling, often chronic, and potentially fatal mental health disorders [4–6]. The global health-related burden of EDs is significant, and in young women it is greater than that of other health conditions [7]. EDs are associated with an increased mortality risk, impaired quality of life, heavy personal and family burden, and high healthcare costs [4–6]. Since the start of the pandemic, many studies have reported rapidly increasing numbers of ED cases, along with deterioration of ED symptoms and psychopathology among those affected [8–16].

Although the pandemic is not yet resolved and its full consequences have yet to be established, our aim is to provide a synopsis of the evidence up to date on the impact of the COVID-19 pandemic on individuals with an ED. 

**Box 1:**
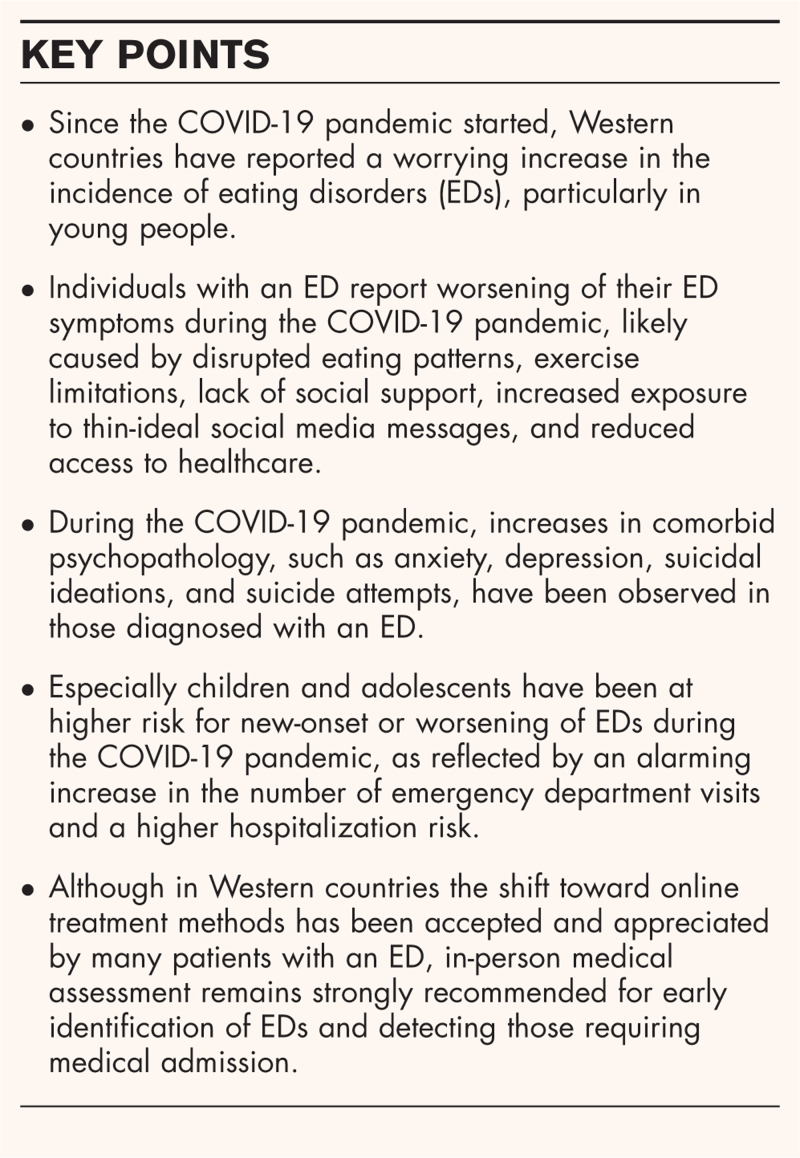
no caption available

## METHOD

A systematic literature search was conducted (via OvidSP) in Medline, Embase, and the Cochrane Database of Systematic Reviews. We used a search strategy with synonyms for ‘COVID-19’ and ‘eating disorder’ and its subcategories (anorexia nervosa (AN), bulimia nervosa (BN), binge eating disorder (BED), pica, rumination disorder, avoidant/restrictive food intake disorder, and other specified feeding and eating disorder (OSFED)). Grey literature and references from bibliographies from relevant papers were inspected for potential eligibility.

Our selection criteria were: (1) peer-reviewed empirical studies and review papers, (2) published in English, German or French, (3) published between January 2020 and May 2022, and (4) that addressed the relation between the COVID-19 pandemic and the number of cases, symptoms, comorbid psychopathology and treatment of EDs. In our results, we primarily focused on studies of patients who were formally diagnosed by healthcare professionals according to DSM-5 or recorded as such in healthcare registers. In case of a lack of such studies, studies involving subjects with a self-reported ED were also considered.

We screened the titles and abstracts to identify eligible articles. Full-text of articles meeting our selection criteria were read before inclusion in this review. The complete search yielded 1394 articles; here we report on 81 articles, including ones providing contextual background. In preparing for this review, the methodological quality of included literature was assessed with either the National Institutes of Health quality assessment tools [17] or the Critical Appraisal Skills Programme checklist [18]. Article selection, data extraction, and assessment of methodological quality were performed by the first author (KM). Any doubts on methodological quality were discussed with the second author (DH). The search strategy used for this review and the quality assessment are available upon request.

## NUMBER OF EATING DISORDERS CASES

We found two population-based studies reporting the incidence of EDs in adults since the start of the COVID-19 pandemic [19]. In a database study on 5.2 million health records from US individuals aged under 30, Taquet *et al.*[20^▪▪^] found that in 2020 the overall incidence of EDs had increased by 15% compared with previous years (RR 1.15, 95% CI 1.12–1.19). Similarly, Asch *et al.*[21] used health records of 3 million US individuals from a commercial insurance company. They showed that the number of ED inpatients remained approximately stable around 0.3 per 100,000 individuals from January 2018 to April 2020 and doubled to 0.6 between May 2020 and December 2020. Likewise, the number of ED outpatients increased from 25 to 29 per 100,000 per month from 2019 to 2020 [21].

## HOSPITAL ADMISSIONS AND EMERGENCY DEPARTMENT VISITS

A systematic review based on 59 cross-sectional studies (prepandemic *n* = 591, in-pandemic *n* = 876) demonstrated an overall average increase of 48% (range 0–123%) in hospital admissions for EDs during the pandemic compared to previous timepoints [22]. The increase of healthcare use in individuals with EDs has been largely observed among women [20^▪▪^], children and adolescents [20^▪▪^,^21^–^24^], and those with AN [20^▪▪^,^21^,^25^,^26^].

Anderson *et al.*[27^▪^] used the National Syndromic Surveillance Program database to evaluate the number of mental-health-related emergency department visits of adults aged 18–64 years at 3600 US medical centers between January 2019 and August 2021. After a peak in COVID-19 cases, ED-related emergency department visit counts increased by 11.1% in the period February-March 2021 compared to the same (prepandemic) period in 2019. Increases in the numbers of emergency department visits were highest in females (12.7%) and in younger adults (aged 18–24 years; 22.8%) with an ED. Also, there was a higher rate ratio (RR) of 1.31 (95% CI: 1.19–1.44) for the number of ED-related versus total number of emergency department visits after the peak compared to prepandemic, indicating an increased risk for an emergency department visit in persons with an ED relative to those without an ED [27^▪^].

## PATHWAYS OF INCREASED RISK

Disruptions and restrictions in daily life during the pandemic are believed to have increased the risk for onset or deterioration of EDs [2,8,10,28^▪^]. Rodgers *et al.*[28^▪^] hypothesized three pathways for how measures to prevent further spread of the virus promoted this risk, which are supported by other studies [11,25,29–32,33^▪^,^34^,^35^]. First, social distancing and imposed lockdowns disrupted daily routines (eating, sleeping, and exercising) and mobility. Consequently, individuals with an ED experienced increased concern from the lack of daily structure, not being able to stick to meal and exercise plans, and spending more time in a triggering environment [11,22,28^▪^,^31^,^32^,33^▪^,^34^,^35^,^36^^▪▪^]. Individuals with an ED reported increased anxiety and depressive symptoms, leading to emotional eating and shape or weight concerns [11,28^▪^,^29^–^32^,33^▪^,^34^]. In addition, limited access to physical exercise (e.g., due to closing of sports facilities) fostered increased levels of anxiety related to inactivity in people with an ED [30,36^▪▪^]. Closing schools, work places, and sport events isolated children and adolescents from their peers, leaving them unable to socialize, train together, and learn in a group setting [8,13]. Furthermore, social isolation deprived patients with an ED of some protective factors, such as social support, security, and adaptive coping strategies [28^▪^,^30^,^31^,^35^,^37^]. Second, the pandemic accelerated the tendency to spend time online, because lack of in-person interactions and social isolation led to increased use of social media platforms, particularly during the lockdowns. This increased the risk of exposure to thin-ideal related content and anxiety-provoking media, increasing the risk for EDs in susceptible individuals [11,22,28^▪^,^34^,^37^]. Similarly, during the pandemic video-telephony was widely used as a medium to communicate. As individuals with an ED are susceptible to preoccupation with their body image and appearance, video-telephony may also increase ED risk [28^▪^]. Third, the pandemic led to increased stress levels, emotional distress, and anxiety among the whole population, which are all key risk factors for EDs [28^▪^].

Overall, disruptions and changes to daily life had the potential to trigger the onset of ED symptoms in some individuals and to worsen them in others. Few studies described positive effects of the pandemic, although in some studies individuals with an ED reported more social support and connectedness with family, an improved structure in daily routines, and a higher motivation to recover [22,34,35,38,39].

## EATING DISORDER SYMPTOMS

When ED symptoms in patients are compared pre and in-pandemic, most studies so far have employed cross-sectional designs and/or involved retrospective recall (e.g., [38,40]). Hence, these studies are prone to bias with respect to the selection of participants and reliability of reported eating problems. Overall, increases in body shape and eating concerns (between 29% and 80%), compensatory behaviors (between 7% and 36%), dietary restriction (between 44% and 65%), exercise behaviors (between 42% and 50%), and binge eating (between 14% and 47%) have been reported [29–32]. Furthermore, Monteleone *et al.*'s systematic review [30] reported a worsening of ED symptom severity in 38–83% in individuals with an ED after the first wave of the pandemic [30]. This was similar for patients with a lifetime ED diagnosis and those with a current ED. In Sideli *et al.*'s meta- analysis [32], most ED patients reported that the pandemic led to a worsening of symptoms. When comparing pre and in-pandemic levels, no differences in weight, symptom severity, and binge-eating symptoms were detected after pooling. Sideli *et al.* noted that due to the range of outcome measures, their analyses were performed on only 15% of eligible papers, which made it difficult to assess any changes [32]. In two meta-analyses published in 2022, pooled prevalences of individuals with an ED who experienced worsening of ED symptoms due to the pandemic were comparable, namely 60% (n = 7848, 95% CI: 49–70%) [36^▪▪^] and 57% (*n* = 963, 95% CI: 36–76%) [41^▪▪^]. The overall prevalence of individuals with improved ED symptoms was 9% (*n* = 741, 95% CI: 4–17%) [36^▪▪^]. Diversity in measured constructs, significant publication bias, and lack of replicability of findings across included studies were reported to lead to uncertainty regarding the relation between symptom changes and the pandemic in individuals with an ED [29,36^▪▪^,^41^^▪▪^].

In response to the need for a uniform outcome measure, the COVID Isolation Eating Scale (CIES) was developed and validated to study the pandemic's impact on eating-related symptoms and psychopathology in individuals with an ED and obesity [39]. It was used to study more than 800 individuals with an ED or obesity in Europe and Asia [33^▪^]. Individuals with AN reported worsening in their eating style and increased alcohol consumption. Those affected with BN reported weight gain, increased alcohol consumption, and emotional dysregulation. Individuals with BED reported weight gain and impaired eating styles. The heterogeneous group of patients with OSFED showed no differences in ED symptoms before and during the pandemic [33^▪^]. Compared to other ED subtypes, individuals with OSFED reported the highest level of psychological impairment, i.e. symptoms of anxiety, depression, and emotion dysregulation, due to the pandemic [33^▪^]. However, as the CIES involved retrospective recall, the reliability of the results was limited by recall bias [33^▪^].

Worsening of core symptoms consistent with ED subtype has been found in other studies too, i.e., binge-eating behaviors were exacerbated in patients with BN, whereas patients with AN expressed greater shape concerns and more compensatory physical exercise [30,38,40,42].

## OVERALL MENTAL HEALTH

Whereas the results of the COVID-19 pandemic's effect on ED symptomatology are mixed, the data on overall mental health are more consistent. Overall, the pooled prevalence of anxiety symptoms and depressive symptoms in patients with an ED during the pandemic were 64% (95% CI: 48–79%) and 55% (95% CI: 12–87%), respectively [41^▪▪^]. Increased symptoms of anxiety, depression, and posttraumatic stress disorder were reported retrospectively in at least 50% of individuals with an ED during the first lockdown compared to both prelockdown [22,30,32,36^▪▪^,^43^,^44^] and to healthy controls in the same period [45]. Patterns of change in pre versus in-pandemic anxiety and depressive symptoms appeared to be sensitive to timing (e.g., worse during lockdowns) [22]. In a population-based study, suicidal ideations and -attempts increased among patients with an ED in 2020 compared to 2019, but suicide death rates remained stable [20^▪▪^]. In Linardon *et al.*[29], 37–80% of patients with an ED reported a worsening of their general mental health, quality of life, and general well-being due to the pandemic. In a systematic review, 62% of individuals with BN and 50% of individuals with AN reported a lower quality of life [30]. Furthermore, 70–75% of people with AN or BN reported more feelings of loneliness, sadness, and inner restlessness [38,40]. Generally, there were insufficient data to determine whether these changes differ between ED subtypes.

## RISK AND RESILIENCE FACTORS

Prepandemic low self-directedness, traumatic experiences, insecure attachment, fear of contagion, and reduced satisfaction with social relationships were found to be associated with in-pandemic worsening of ED symptoms [30,46,47,48^▪^]. Sideli *et al.*[32] identified social isolation and stress caused by COVID-19 as risk factors for worsening of ED symptoms and weight gain during the first lockdown. Changes in routine, exposure to thin-related social media messages, social isolation, and negative emotions (e.g., heightened rumination, anxiety) were indicated as risk factors for poorer mental health in general [30,34]. Factors that protected from deterioration in mental health were heightened self-directedness [30], less pressure to participate in social situations or work [30], social and family support [32], and capacity to maintain goals and perceived control [32].

Mental health disorders have been associated with increased COVID-19-related mortality [49]. Few studies to date have examined the severity and lethality of COVID-19 infections in individuals with an ED. Generally, both underweight and obesity can increase viral infection risk in a U-shaped relation with body mass index (BMI) [50]. As symptoms and laboratory findings during COVID-19 infections are similar to those found in patients with an ED (especially AN), detection and surveillance of COVID-19 infections in those with an ED may be complicated [51,52]. However, it has been hypothesized that AN patients are less prone to viral infections than individuals with malnutrition [52]. In one small observational study, having AN did not increase the risk of severe COVID-19 infection [53]. Further studies are needed to assess the susceptibility to COVID-19-related complications and mortality among patients with an ED.

## CONCERNS ABOUT CHILDREN AND ADOLESCENTS WITH AN EATING DISORDER

Mental health problems in young people with an ED were a matter of serious concern during the pandemic [3^▪▪^,^8^,^9^]. This is illustrated by a report from the US Centers for Disease Control and Prevention (CDC), which showed that pediatric emergency department visits for mental health conditions increased by 24% among children and by 31% among adolescents between March and October 2020 compared to the same period in 2019 [3^▪▪^]. In general, 42–81% of children and adolescents with an ED experienced worse ED symptoms in the pandemic [54,55], and also more depressive symptoms, anxiety and suicidal ideations were reported by young people with an ED during the first pandemic wave [25,54,56]. In a Canadian study, children and adolescents admitted for an ED to a pediatric tertiary care center had worse symptom severity during the pandemic compared to prepandemic (e.g., excessive exercise, purging, eating restraint) [56].

The exacerbation of symptoms in young people with an ED was noticed in many healthcare systems. The CDC calculated the change in average weekly emergency department visits and proportion of visits for EDs among young people below 18 years old in the US. They found that for 12–17-year-old females weekly visits for EDs increased by 60% during 2021, and by 64% in early January 2022, compared with 2019, as did the proportion of visits for an ED in 2020, 2021 and January 2022 compared to 2019 (RR: 1.95, 2.29, and 1.99, respectively) [3^▪▪^]. Various studies in Canada reported similar results. A Canadian population-based study showed that since the start of the pandemic the risk for acute care visits for EDs increased by 66% (RR 1.66, 95% CI 1.41–1.96) and for hospitalization by 37% (RR 1.37, 95% CI 1.25–1.50) [57^▪▪^]. Another Canadian cross-sectional study in six tertiary care pediatric hospitals showed an increased incidence of de novo AN or atypical AN diagnoses from on average 24.5 to 40.6 cases per month after the first wave of the pandemic (November 2020) [58^▪▪^]. At the same time, hospitalizations for newly diagnosed AN or atypical AN increased from 7.5 to 20.0 cases per month [58^▪▪^]. Another Canadian study showed that children and adolescents with EDs more often both were medically unstable at admission and required medical admission in 2020, compared to 2019 [56]. In the same period, urgent outpatient referrals for an ED increased by 56% in this center [56]. In a US pediatric medical center, readmission within 30 days occurred nearly nine times more often for children or adolescents with AN or atypical AN during lockdown compared to prepandemic [59]. Several other single-center studies in pediatric hospitals in Australia, US, Canada, New Zealand, and Israel reported significant increases in hospital admissions, emergency department visits, hospitalization risk, and outpatient referrals for ED in children and adolescents during the pandemic compared to prepandemic [23,25,60–63].

## ONLINE TREATMENT

Because access to in-person treatment and primary care was limited, alternatives for in-person treatment were quickly introduced at the start of the pandemic in order to maintain continuity and accessibility of care [64]. We refer to ‘online treatment’ as real-time, remote clinical services between healthcare providers and patients, including video-telephony. Several small, uncontrolled studies (*n* = 4–87) [39,55,65–72] and one controlled study (*n* = 125) [73] reported that online treatment has been accepted and is appreciated by most ED outpatients as an alternative for in-person therapy. In total, 67–71% of ED outpatients reported online treatment to be better than, or as good as, in-person treatment provided in the prepandemic situation [55,65]. Other reported positive aspects of the shift to online treatment were easy treatment accessibility, being around family, and flexibility [66–68]. Being satisfied with the online treatment was associated with higher anxiety levels related to COVID-19, longer illness duration, and a stronger therapeutic relation with the care provider [69].

The clinical effectiveness of online treatment during the pandemic, compared to in-person care prepandemic, was examined in uncontrolled (*n* = 55–159) [38,40] and small, nonrandomized controlled studies (*n* = 9–365) [65,73–76]. Although the results are preliminary, the switch to online treatment as an alternative for in-person treatment was found to be as effective, safe, and well-tolerated as face-to-face treatment in most studies in outpatients [65,73–75,77^▪^]. In most studies, clinical effectiveness on weight gain and psychopathology was found to be similar before and after the switch to online treatment [73–75]. One controlled study observed that face-to-face day treatment for patients with AN resulted in higher rates of weight restoration in comparison to online day treatment [76].

The increased use of online treatment was accompanied by concerns about treatment quality [38,40,67,69,72,77^▪^]. In total, 56% of patients would not recommend online treatment to others [69], but only less than 10% said they would prefer not to continue using it after the end of the COVID-19 pandemic [66,69]. Some ED patients had concerns about self-monitoring, sensed that aspects of communication were lost, or felt less pressure to resist their illness [30,39,66,67]. Body image concerns, technological difficulties, and lack of privacy reduced satisfaction with online treatment [30,32]. Those with limited access to online services (e.g., because of unfamiliarity or unavailability) and those living in middle- and low-income countries are currently being overlooked – both in studies and in online treatment options [64,78^▪^,^79^]. None of the studies reported on experiences with online therapy prior to the pandemic.

Treatment provider perceptions of online therapy were mixed [77^▪^]. During the pandemic, healthcare professionals were able to creatively adapt treatments to an online treatment format. Online treatment was found to be feasible and easily scaled-up. Some treatment providers reported feelings of isolation, and less therapeutic alliance and quality of care [77^▪^].

Recently, a consensus paper was published with recommendations for online treatment of patients with an ED during the pandemic [78^▪^]. The authors strongly recommend maintaining in-person evaluations to assess medical instability that requires hospital admission. As an alternative for in-person therapy, ‘internet-based cognitive behavioral therapy (CBT) guided self-help’ was strongly recommended in emerging adults (18–25 years) with an ED. The CBT self-help parental guide was recommended for caregivers of individuals with an ED. In children and adolescents with an ED, there was only weak evidence for online treatment methods. ‘Telehealth family-based treatment (FBT)’ and ‘online guided parental self-help FBT’ were supported as weak recommendations [78^▪^]. Guidelines for adults with an ED have not yet been established.

## QUALITY CONSTRAINTS

The nascent field of research on the impact of COVID-19 on EDs is evolving rapidly. However, the wide array of studies of varying quality hinders the interpretability of results for this and other reviews (e.g., [29,32]). The studies identified by our search strategy comprised a large body of literature based on individuals with a self-reported ED. To increase the representability of the results, for this review, we chose to focus on studies on ED patients clinically diagnosed by healthcare professionals, or recorded as such in health registers. We highlighted the results from the small share of longitudinal studies and/or controlled studies.

Most studies reported that the pandemic has had negative effects on individuals with an ED, but the heterogeneity in outcome measures makes it difficult to assess the true impact of the COVID-19 pandemic. The majority of studies employed a cross-sectional design and involved retrospective recall, making them prone to participants’ recall bias. Moreover, most of the study populations in the identified studies consisted of Caucasian females, young participants, and/or individuals diagnosed with AN. The literature largely fails to address the situation in other than Western countries.

## CONCLUSION

It is difficult to imagine a global event that promotes as many risk factors for EDs as the current COVID-19 pandemic. The social isolation, disruption of routines, exercise and habits, increased online media usage, and the fear caused by the pandemic were all likely to increase the risk of onset of an ED, or worsen symptomatology in those already affected. Indeed, symptoms of anxiety, depression, posttraumatic stress disorder, and burn-out have been more frequently reported by individuals with an ED during the pandemic and its various lockdowns, compared to prepandemic. Since the start of the pandemic (in early 2020 in Western countries), the incidence of EDs has greatly increased and this is most apparent in young individuals. Alarming increases, with growing numbers of inpatients, outpatients, referrals, and emergency admissions in 2021 and early 2022, have been reported among children and adolescents with an ED. Newly admitted young patients with an ED were more often medically unstable and more often required medical monitoring and readmission. The worse clinical condition of young people with an ED at presentation during the pandemic is reason for concern, particularly given the reduced access to face-to-face healthcare during the pandemic.

The shift toward online treatment, although still in its infancy, has been accepted and appreciated by many ED outpatients. Healthcare providers have used various approaches to offering online treatment. To date, internet-based CBT-guided self-help is recommended for emerging adults. Despite social distancing measures, in-person medical assessment remains strongly recommended in the care for EDs, since they are essential for early identification of patients with an ED and those with medical instability requiring hospital admission.

It is still too early to conclude whether the impact of the pandemic will be temporary or longer-lasting after lockdown measures are lifted, and after reopening work- and social life. Many Western countries have removed restrictions on travel-, work- and social life in the spring of 2022. In May 2022, around 66% of people worldwide received at least one COVID-19 vaccine dose [80]. As transmission rates remain high, the emergence of a new COVID-19 variant seems inevitable. Healthcare systems need to strengthen capacities and anticipate an increase in referrals and admissions for mental health disorders, such as EDs [81].

The rapid expansion of research into EDs and the COVID-19 pandemic implies that new reports will soon become available. It still needs to be established why the increase in ED cases has mainly affected young people. This makes more research on EDs in relation to the COVID-19 pandemic imperative, especially given the serious consequences of EDs [4–7]. We underscore the need for more longitudinal, high-quality research with uniform outcome measures on the consequences of the COVID-19 pandemic in individuals with, or at risk for, an ED. There is a need for long-term prospective studies that include non-Western, non-Caucasian, and male populations. Lastly, we also call for high-quality research on populations with limited access to online treatment.

## Acknowledgements

*We thank Franka Steenhuis for assistance with the literature search.*

### Financial support and sponsorship

*No specific funding was received for this work.*

### Conflicts of interest

*There are no conflicts of interest.*

## REFERENCES AND RECOMMENDED READING

Papers of particular interest, published within the annual period of review, have been highlighted as:

▪ of special interest
▪▪ of outstanding interest
